# Cardioembolic stroke related to limb-girdle muscular dystrophy 1B

**DOI:** 10.1186/1756-0500-6-32

**Published:** 2013-01-29

**Authors:** Chih-Hao Chen, Sung-Chun Tang, Yi-Ning Su, Chih-Chao Yang, Jiann-Shing Jeng

**Affiliations:** 1 Department of Neurology, National Taiwan University Hospital, No. 7 Chung-Shan South Road, Taipei, Taiwan; 2 Department of Medical Genetics, National Taiwan University Hospital, Taipei, Taiwan

**Keywords:** Neuromuscular disorder, Limb-girdle muscular dystrophy, Emery-Dreifuss muscular dystrophy, Cardioembolic stroke, Thrombolytic therapy, Cardiac arrhythmia

## Abstract

**Background:**

Cardioembolic stroke is an under-recognized complication in patients with limb-girdle muscular dystrophy 1B. Here we present a young stroke patient who had a novel lamin A/C gene (*LMNA*) mutation.

**Case presentation:**

This is a 39-year-old man who had slowly progressive proximal muscle weakness and cardiac arrhythmia since adolescent and a family history of similar manifestation. He sustained acute ischemic stroke in the left middle cerebral artery territory. Intravenous recombinant tissue plasminogen activator therapy was given with significant neurological improvement. Additionally, genetic sequencing of the *LMNA* gene of the patient identified a mutation in c.513+1 G>A that resulted in a splicing aberration.

**Conclusion:**

We suggested that *LMNA* gene related myopathies should be considered in young stroke patients with long-standing myopathic features.

## Background

Limb-girdle muscular dystrophy 1B (LGMD1B) is an autosomal dominant muscular dystrophy caused by a lamin A/C gene (*LMNA*) mutation, and is characterized by slowly progressive proximal weakness with few contractures and age-related cardiac arrhythmias. The *LMNA* gene is located on chromosome 1q21.1–21.2 and comprises 12 exons consisting of a 25 KB coding region of the gene [1,2]. The *LMNA* gene encodes lamins A and C, which are components of the nuclear envelope but are located in the lamina, a multimeric structure associated with the nucleoplasmic surface of the inner nuclear membrane. Lamins are structurally homologous with other intermediate filaments, and are expressed in a wide range of tissues, including adult heart and skeletal muscle [3].

Mutations of the *LMNA* gene are associated with multiple allelic diseases with widely varying phenotypes, including autosomal dominant dilated cardiomyopathy with conduction defects (DCM-CD), AD-EDMD, LGMD1B, autosomal recessive Charcot-Marie-Tooth disease type 2, mandibuloacral dysplasia, familial partial lipodystrophy, Hutchinson–Gilford progeria, and atypical Werner syndrome (Table 1) [4-6] The skeletal muscle phenotypes of laminopathy, namely autosomal dominant Emery-Dreifuss muscular dystrophy (AD-EDMD) and LGMD1B, are overlapping syndromes exhibiting greater or lesser degrees of muscle weakness, joint contractures and cardiac dysfunction [7,8]. Dilated cardiomyopathy with conduction defects and arrhythmias requiring pacemaker placement affect both groups of patients. Thus, cardioembolic stroke is a severe but rarely reported complication [4].

**Table 1:** **List of important diseases caused by mutations of the *LMNA *gene**

**Disease (and its acronym)**	**Inheritance pattern**
**Striatal muscle involvement**	
Emery-Dreifuss muscular dystrophy (AD-EDMD)	AD
Limb-girdle muscular dystrophy 1B (LGMD-1B)	AD
Dilated cardiomyopathy and conduction-system disease (DCM-CD)	AD
**Peripheral neuropathy**	
Charcot-Marie-Tooth disease type 2B1 (CMT-2B1)	AR
**Partial lipodystrophy syndrome**	
Familial partial lipodystrophy, Dunnigan type (FPLD2)	AD
Mandibuloacral dysplasia I (MAD1)	AR
**Premature aging**	
Hutchinson–Gilford progeria syndrome (HGPS)	AD
Atypical Werner’s syndrome (AWRN)	AD

*AD* autosomal dominant, *AR* autosomal recessive.

Here we present a case of a young stroke patient with long lasting myopathic features which prompted the detection of a novel *LMNA* mutation. Intravenous administration of recombinant tissue plasminogen activator (rt-PA) successfully improved his initial severe neurological disability.

## Case presentation

The patient was a 39-year-old man, who was the first of 2 siblings. His mother had a history of slowly progressive proximal muscle weakness, received pacemaker implantation for slow heart rate at the age of 40 years, and died from sudden cardiac arrest at the age of 50. His younger brother also had similar proximal lower limbs weakness; however he committed suicide at age 31 years. For our index patient, toe-walking and frequent falls were first noted at the age of around 5 years. In his adolescence, he developed proximal leg weakness with Gowers’ sign (i.e., with hip girdle weakness, the patient arises from a stooped or a squatting position by using his hands to “climb up the legs”). Around age 30 years, atrial fibrillation (AF) was found, and he was prescribed with aspirin and an angiotensin-converting enzymes inhibitor. In addition, mild ankle contractures were noted. An electromyographic study revealed myopathic changes, consisting of muscle potentials of small amplitudes and polyphasic waves of short duration, in biceps brachii and rectus femoris. His baseline creatine kinase level was around 200 IU/L (reference range, <190 IU/L for a male). At age 36 years, he complained of chest tightness, and electrocardiography showed AF with slow ventricular rate, right bundle branch block, and transient ventricular tachycardia. Pacemaker implantation (VVI pacemaker, i.e., ventricular pacing, ventricular sensing, pacemaker inhibited) was done at that time.

**Figure 1:**
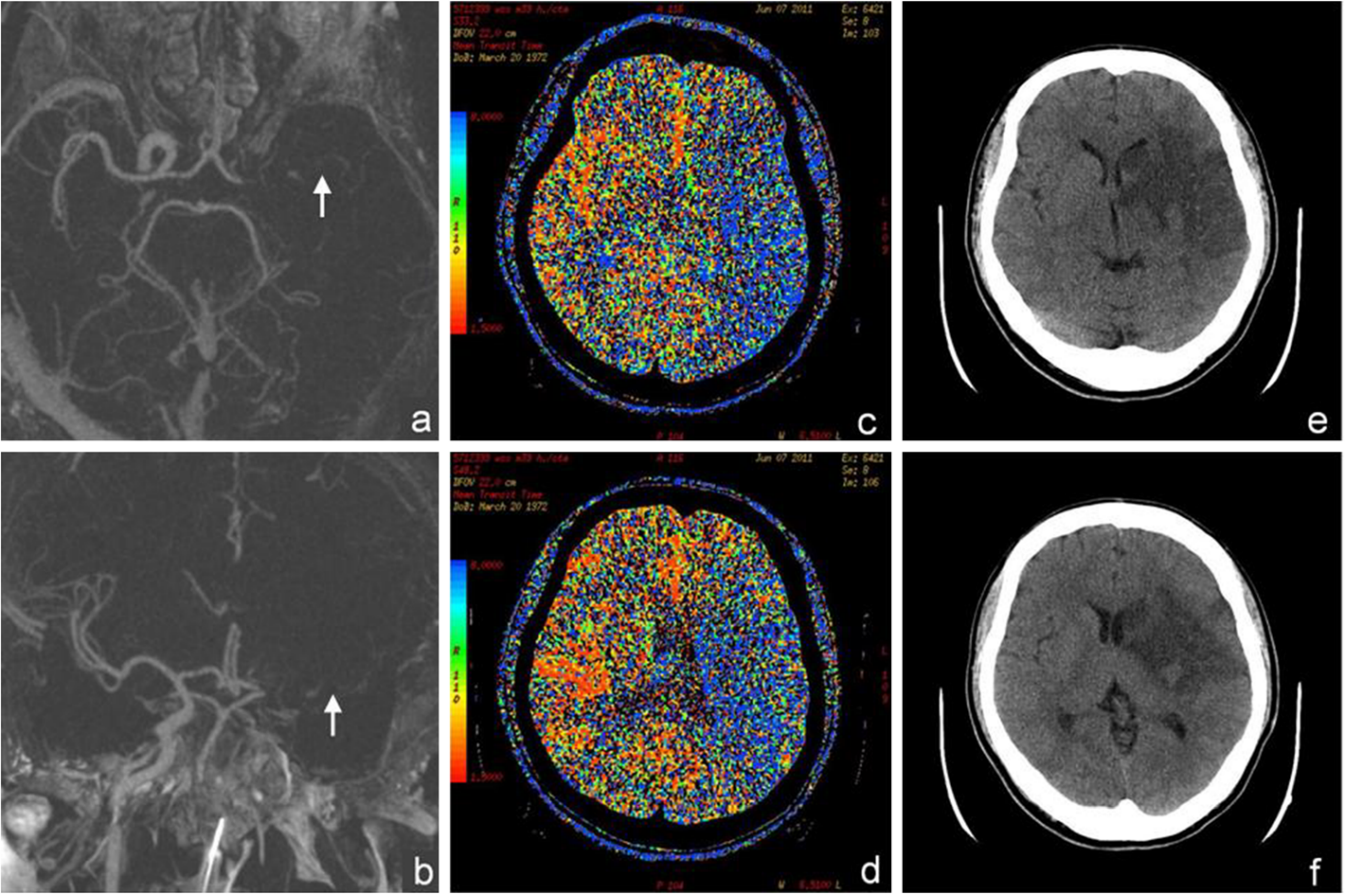
**CT imaging of the patient. **(**a**, **b**) Reconstructed CT angiography revealed nearly total occlusion of left proximal middle cerebral artery (marked by the arrows). (**c**, **d**) CT perfusion study showed prolonged mean transit time (the blue area indicates the area of prolonged transit time) in most of the left cerebral hemisphere, which indicated reduced perfusion. (**e**, **f**) Follow-up non-contrast CT one day after the stroke showed a hypodensity in the left basal ganglia and peri-sylvian area, suggestive of a recent infarct. The size was much smaller than the previous perfusion defect.

He suffered from acute onset of right limb weakness and speech disturbance. The acute onset of focal neurological symptoms was suggestive of stroke, thus he was sent to our hospital immediately. Neurological examination revealed global aphasia, right hemianopia, gaze deviation to the left side, and right hemiplegia. The National Institutes of Health Stroke Scale (NIHSS) score was 22. Initial head computed tomography (CT) did not reveal intracranial hemorrhage. He received intravenous thrombolytic therapy of recombinant tissue plasminogen activator (rt-PA) (73 mg, equal to 0.9 mg/kg) within 3 hours of stroke onset. CT angiography right after thrombolytic therapy revealed nearly total occlusion of the left distal internal carotid artery and proximal middle cerebral artery (MCA) (Figure 1a and b), and CT perfusion image revealed reduced cerebral perfusion in the left hemisphere (Figure 1c and d). Follow-up head CT on the next day showed a hypodensity lesion mainly in the left MCA territory (Figure 1e and 1f), and the size was much smaller than the previous perfusion defect. Transcranial color-coded sonography showed a visible MCA flow signal on color mode but mildly reduced flow velocity on Doppler mode, indicating partial recanalization. Two-dimensional echocardiography revealed a left ventricular ejection fraction of 47%, and enlargement of bilateral ventricles and left atrium. Twenty-four hours after thrombolytic therapy, his NIHSS score was 16; one week later the NIHSS score significantly improved to 5. He was treated with an oral anticoagulantion for secondary stroke prevention. After rehabilitation, his right hemiplegia and aphasia improved gradually. Six months after stroke, he could ambulate without assistance, and speak fluently with only mild naming difficulty.

**Figure 2:**
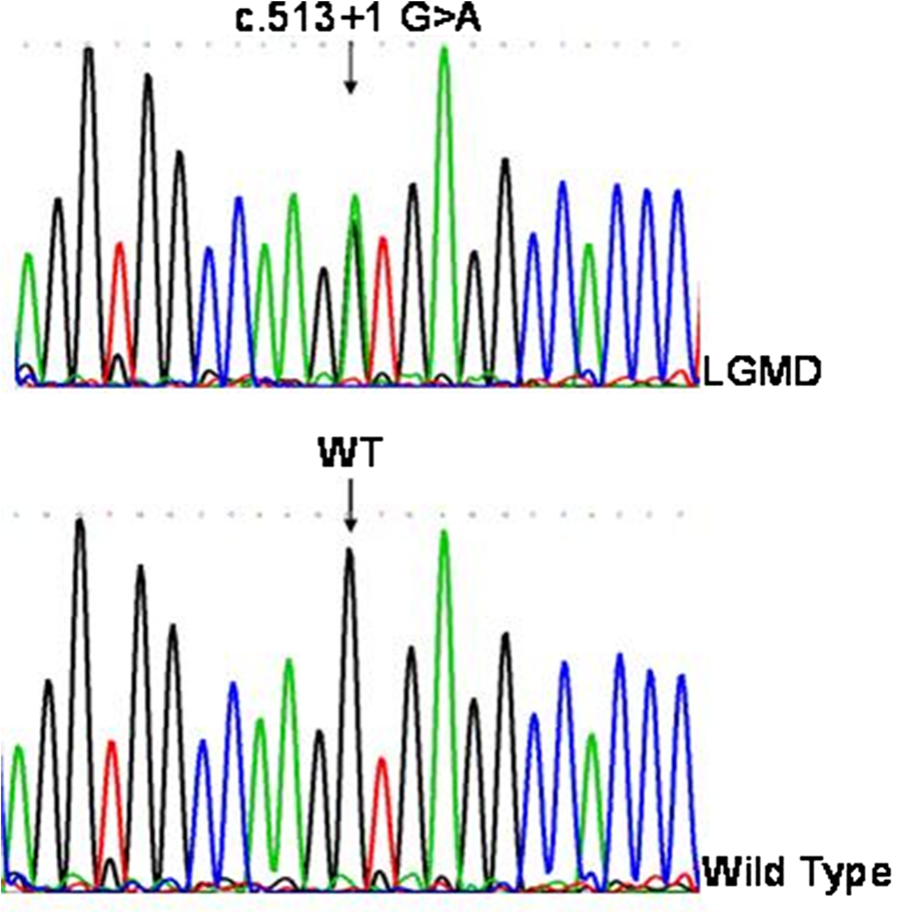
**The results of genetic sequencing of the *LMNA *gene.** In this electropherogram, the green line represents adenine (A), the blue line represents cytosine (C), the black line represents guanine (G), and the red line represents thymine (T). Compared with the wild type (WT, the lower part), our patient carries a heterozygous mutation of “G” to “A” transition at intron 2 (c.513+1 G>A, the upper part).

Given the myopathic features with mild contracture, cardiac arrhythmia and dilated cardiomyopathy, and also positive family history, *LMNA* related muscular dystrophies was highly suspected. For further genetic analysis, the patient’s peripheral blood was obtained with informed consent. All the coding and flanking regions of the *LMNA* gene were amplified by polymerase chain reaction with primers. The confirmatory sequencing demonstrated a heterozygous mutation of “G” to “A” transition at the splicing donor site at intron 2 (c.513+1 G>A / wild type, Figure 2). The nucleoside change was not identified in screening 50 Taiwanese controls. All the study subjects had signed informed consent and the study protocol was approved by the National Taiwan University Hospital ethics board committees.

## Discussion and conclusions

Mutations in the *LMNA* gene cause a variety of human disease with numerous different phenotypes [4-6]. The skeletal muscle phenotypes of AD-EDMD and LGMD1B overlap. Both diseases are inherited in an autosomal dominant fashion and express as slowly progressive proximal limb weakness. AD-EDMD has more prominent muscle wasting in humero-peroneal distribution, early contractures of the elbow flexors, Achilles tendons and rigid cervical spine, and has lethal cardiac arrhythmia that requires pacemaker insertion. The cardiac involvement of AD-EDMD may occur at any age or even as an isolated presentation without detectable skeletal muscle manifestation [8]. On the other hand, LGMD1B has typical girdle weakness, absent or minimal contractures, and has age-related severity of atrioventricular conduction disturbances. The neuromuscular symptomatology almost always precedes cardiological involvement in LGMD1B [9]. Therefore, our patient should be classified as having LGMD1B based on less contractures and age-related cardiac conduction disturbance.

Ischemic stroke of cardioembolic origin is a relatively rare but severe complication among patients of myopathy and cardiac arrhythmia. In a literature review, the prevalence of AF or atrial flutter in patients with primary myopathies was estimated as 15% [10]. The most frequently reported myopathies that are associated with arrhythmia included myotonic dystrophy, EDMD, dystrophinopathies, and limb girdle muscular dystrophies. The stroke rate in patients with myopathy and AF/atrial flutter was around 6.5%, and most of them received oral anticoagulant for secondary stroke prevention [10].

Limited data are available about the incidence of stroke in laminopathy, namely AD-EDMD or LGMD 1B. We reviewed the published literature and found 7 previously reported cases (Table 2) [4,11-14]. All are ischemic strokes, and all except our patient are characterized by the AD-EDMD phenotype. Most of the patients suffered from stroke at a relatively young age (less than 45 years old). All had documented cardiac arrhythmias (usually AF and atrioventricular conduction block), and many of them had received pacemaker or implantable cardioverter-defibrillator (ICD) insertion. Thus, although no current guidelines are available, oral anticoagulant is advisable in such cases with AF for stroke prevention. Pacemaker or ICD implantation should be considered as soon as bradycardia or ventricular tachyarrhythmia occurs, even as a primary prevention method, to reduce the risk of sudden death [15].

**Table 2:** **Comparison of patients with *LMNA *gene mutation and stroke**

**Author**	**Age of stroke**	**Stroke signs**	**Stroke location**	**Arrhythmia during all follow-up**	**Pacemaker insertion and age**	**Phenotype**	**Mutation of *LMNA *gene**
Onishi et al., 2002 [11]	45	Right hemiplegia	N/A	AF, AVB, VPC	Pacemaker, 45	AD-EDMD	p.Ser303Pro (c.907 T>C)
Boriani et al., 2003 [4]	26	Hemiplegia	N/A	AF, AFL, AVB	VVI, 30	AD-EDMD	p.Arg386Lys (c.1157 G>A)
	57 and 70	N/A	N/A	AF, AVB, SAB	VVI, 55	AD-EDMD	p.Arg527Pro (c.1580 G>C)
	43 and 44	Ataxia	Cerebellum	AF, AVB	VVI, 41	AD-EDMD	p.Arg377Leu (c.1130 G>T)
Liang et al., 2007 [12]	45	Conscious loss	Pons	VPC, VT, VF	nil	AD-EDMD	p.Trp520Gly (c.1558 T>G)
Redondo-Vergé et al., 2011 [13]	25	Aphasia, right hemiparesis	Left MCA	AF, AVB	ICD, 25	AD-EDMD	p.Arg89Leu (c.266 G>T)
Tanaka et al., 2012 [14]	12	Dysarthria, left hemiparesis	Right MCA	AF	nil	AD-EDMD	N/A
Present case	39	Aphasia, right hemiplegia	Left MCA	AF, RBBB, AVB	VVI, 37	LGMD1B	c.IVS2+1 G>A (c.513+1 G>A)

*AF* atrial fibrillation, *AVB* atrioventricular block, *ICD* implantable cardioverter defibrillator, *MCA* middle cerebral artery, N/A non available, *SAB* sinoatrial block, *VPC* ventricular paroxysmal contraction, *VT* ventricular tachycardia.

To date, more than 300 mutations have been reported in *LMNA* gene (based on an on-line database: http://www.UMD.be/LMNA/). A novel mutation in c.513+1 G>A was identified from our index patient. Although the mutation does not affect the coding region, its position is in the splice donor site of intron 2. We speculate this splicing donor site mutation may be associated with abnormal splicing process. One German pedigree was reported to have a synonymous codon change of *LMNA* gene in c.513 G>A, resulting in a so-called “neutral” mutation of Lys171Lys, which leads to abnormal splicing of intron 2 and possibly causes LGMD1B [16]. In addition, another family of LGMD1B had a mutation in the splice donor site of intron 9 (IVS9+5 G>C), which also leads to abnormal splicing of *LMNA* mRNA [17]. Therefore, our case probably had a disease-causing mutation in the intron, which leads to abnormal splicing of *LMNA* gene and mutant lamin production.

In conclusion, we presented a LGMD1B patient with proven *LMNA* gene mutation who suffered from disabling cardioembolic stroke, and was treated effectively with intravenous thrombolytic therapy. When a suspected cardioembolic stroke occurring at a relatively young age is associated with long-standing myopathic features, *LMNA* gene related disorders should be considered.

## Consent

Written informed consent was obtained from the patient and his wife for publication of this Case report, any accompanying images, and the included family information. A copy of the written consent is available for review by the Series Editor of this journal.

## Abbreviations

LMNA: Lamin A/C gene; LGMD 1B: Limb-girdle muscular dystrophy 1B; AD-EDMD: Autosomal dominant Emery-Dreifuss muscular dystrophy; rt-PA: Recombinant tissue plasminogen activator; AF: Atrial fibrillation; NIHSS: National Institutes of Health Stroke Scale; CT: Computed tomography; MCA: Middle cerebral artery; ICD: Implantable cardioverter-defibrillator.

## Competing interests

The authors declare that they have no competing interests.

## Authors’ contribution

CHC, CCY, and JSJ examined and evaluated the patient. YNS carried out the molecular genetic studies. CHC drafted the manuscript. SCT and JSJ participated in the design of case-report and helped to draft the manuscript. All authors read and approved the final manuscript.
